# Evaluation of the implementation fidelity of the seasonal malaria chemoprevention intervention in Kaya health district, Burkina Faso

**DOI:** 10.1371/journal.pone.0187460

**Published:** 2017-11-29

**Authors:** Rachidatou Compaoré, Maurice Wambi Evariste Yameogo, Tieba Millogo, Halima Tougri, Seni Kouanda

**Affiliations:** 1 Institut de Recherche en Sciences de la Santé (IRSS), Ouagadougou, Burkina Faso; 2 Institut Africain de Santé Publique (IASP), Ouagadougou, Burkina Faso; Institut de recherche pour le developpement, FRANCE

## Abstract

**Background:**

Burkina Faso implemented the seasonal malaria chemoprevention (SMC) in 2014 in seven pilot health districts, following the new recommendation by the WHO in 2012 for the prevention of the disease in children under five years old, for areas of highly seasonal malaria transmission.The objective of this study was to assess the implementation fidelity of the seasonal malaria chemoprevention strategy in one of the districts, Kaya Health District.

**Methodology:**

We conducted a case study, with a quantitative and qualitative mixed methods. Data were collected after two campaigns of implementation of the intervention, in 2014 and 2015, through a review of specific documents of SMC intervention, and individual interview with key informants (n = 21) involved at various levels in the implementation of the strategy and a household survey with the parents (n = 284) of eligible children for the SMC strategy in 2015 in the Kaya health district. The analysis framework focused on the fidelity of the intervention’s content, its coverage, and its schedule, as well as the potential moderating factors, using the model proposed by Hasson, originally from Carroll.

**Results:**

All components of the intervention were implemented. Villages and sectors were covered at 100%. In terms of intervention doses received, less than one-third of eligible children (32.3%) received the recommended four doses in 2015. Implementation of the strategy faced some difficulties due to insufficient training of community distributors, inadequate supply of inputs and insufficient financial resources for remuneration, advocacy and supervision, but also because of the contextual constraints due to the rainy season. Moreover, an interaction between the different moderating factors, influencing the degree of implementation of the strategy was noted.

**Conclusion:**

Taking into account the moderating factors of the implementation is necessary for achieving the highest possible degree of implementation fidelity and then, reach the expected beneficial effects.

## Background

Malaria remains a major public health problem worldwide and in sub-Saharan Africa in particular. More than two billion people worldwide are estimated to suffer from malaria, which had an estimated morbidity of 216 million clinical cases and caused nearly 655,000 deaths worldwide in 2010 [1]. Approximately 85% of malaria cases and 90% of malaria deaths occur in sub-Saharan Africa, where the vast majority of cases and deaths occur in young children [2].

The interventions currently recommended by the World Health Organization (WHO) for the control of malaria include insecticide-treated nets (ITNs) and/or indoor residual spraying (IRS) for vector control, as well as prompt access to diagnostic testing of suspected malaria and treatment of confirmed cases [3]. Other interventions are also recommended for specific high-risk groups in areas of high transmission and include intermittent preventive treatment in pregnant women and infants [4].

The WHO currently recommends a new intervention to combat *Plasmodium falciparum* malaria entitled Seasonal Malaria Chemoprevention (SMC), which is defined as "the intermittent administration of full treatment courses of an antimalarial medicine during the malaria season to prevent malarial illness with the objective of maintaining therapeutic antimalarial drug concentrations in the blood throughout the period of greatest malarial risk” [3].

The economic feasibility and effectiveness of SMC have been widely demonstrated in several experimental studies that closely controlled the implementation conditions [5–9]. However, there is a lack of evidence and experience regarding the routine implementation of SMC. Indeed, analysing the implementation process and its fidelity is essential to understanding the specific reasons that an intervention succeeds or fails [10–13]. Implementation fidelity is often defined as the degree to which a particular programme follows the original programme model, that is, the model that was intended to be used by the programme developers [14]. Fidelity is especially relevant for complex interventions in settings that are not closely controlled [15, 16]. Indeed, it is well known that effectiveness can be severely limited by an implementation that does not adhere to the intended plans, especially in programmes aiming to reach high levels of coverage when scaling them up [17]. For example, a systematic review showed that the effectiveness of health programmes delivered at scale is currently only about half the effectiveness observed under optimal conditions [18].

Although an increasing number of studies are evaluating the implementation of health programmes according to their intended plans, few have addressed the scale-up of interventions [17, 19–21]. These types of studies are necessary to explain the success or failure of public health prevention and promotion programmes. Thus, the implementation of interventions should be evaluated in their actual context.

Our study aimed to evaluate the implementation fidelity of the SMC strategy in Kaya health district, Burkina Faso, after two yearly campaigns. Specifically, our evaluation addressed several dimensions of the SMC implementation fidelity: adherence to the content and schedule of activities in the initial implementation plan, coverage of planned activities and of the targeted population of children, and identification of possible moderating factors that may affect the outcomes of the intervention [13, 22].

## Methodology

### Study site

Our study was conducted in the north-central region of Burkina Faso in the health district of Kaya, which was one of seven pilot health districts included in the SMC implementation in 2014.

Kaya health district covers 342 villages and seven urban areas and includes 54 Health and Social Promotion centres (CSPS), which are primary health care centres.

The Kaya Health and Demographic Surveillance Site (Kaya HDSS), a research platform established in 2007 by the Institute for Health Science Research (IRSS), is located in this health district. The Kaya HDSS is an INDEPTH Network member centre that conducts regular follow-up household surveys of approximately 70,000 people living in both urban and rural areas of Kaya health district. Further details on the site and the follow-up procedures are available elsewhere [23].

### Description of the intervention

In Burkina Faso, the SMC intervention is implemented during the high malaria transmission period from late July/early August to late October/early November.

#### 1. Drug distribution

SMC is delivered each year to eligible children aged 3–59 months through a four-month drug distribution campaign that provides amodiaquine + sulfadoxine-pyrimethamine (AQ+SP).

Paired community distributors (CDs) dispense a full course of SMC drugs each month to targeted children using a door-to-door approach under the supervision of formal health workers. Each monthly drug distribution or SMC cycle lasts over 4 days, with an interval of exactly one month between two cycles.

A complete course of SMC comprises a single treatment with SP and three daily doses of AQ; the CDs provide the first dose of AQ with SP to each 3- to 59-month-old child under directly observed treatment (DOT) in the absence of any contraindications. The drugs are crushed and mixed with sugary water to mask the bitterness of AQ and to improve drug intake. Children are observed for 30 min after treatment. A second treatment is given if the child vomits or regurgitates the drug within this period. During this period, CDs should also advise children’s caregivers on how to administer the second and third doses of AQ to the child at home and inform them about possible side effects.

Parents of target children who receive the treatment should be provided with an SMC administration card; CDs mark the first doses on this card, and the second and third doses of AQ are marked by the child caregiver.

To maximize protection and to minimize selection of drug resistance, eligible children should receive a complete SMC treatment course each month during the peak transmission period.

#### 2. Other components of the strategy

Before each campaign, the selected CDs are trained on the contraindications of the drugs and how to deliver the intervention and complete the necessary tools. Training of the formal health workers focuses on the use of pharmacovigilance forms, management of breakthrough cases, supervision of CDs and data management.

Input supply and supply chain management, advocacy and community mobilization are activities that should be conducted at the beginning and throughout each SMC cycle.

The head nurse (ICP), who is the chief nurse responsible of each CSPS, transmits daily data on the number of children covered to the health district data managers to estimate the coverage of eligible children per cycle.

### Study design

We conducted a case study, using a quantitative and qualitative mixed-methods design. To evaluate the fidelity of the implementation of the SMC strategy, we used the model proposed by Hasson [22], which was adapted from the one initially proposed by Carroll’s *Conceptual Framework for Implementation Fidelity* [13]. The initial framework includes components of implementation fidelity and factors that may influence the degree of fidelity, referred to as moderating factors. Implementation fidelity measures adherence and its subcategories–content, frequency, duration, and coverage (dose)–as well as potential moderators including intervention complexity, participant responsiveness, strategies to facilitate implementation, and quality of delivery. We added the context of implementation, one of the two new moderating factors proposed by Hasson [22], to this model.

**Fig 1:**
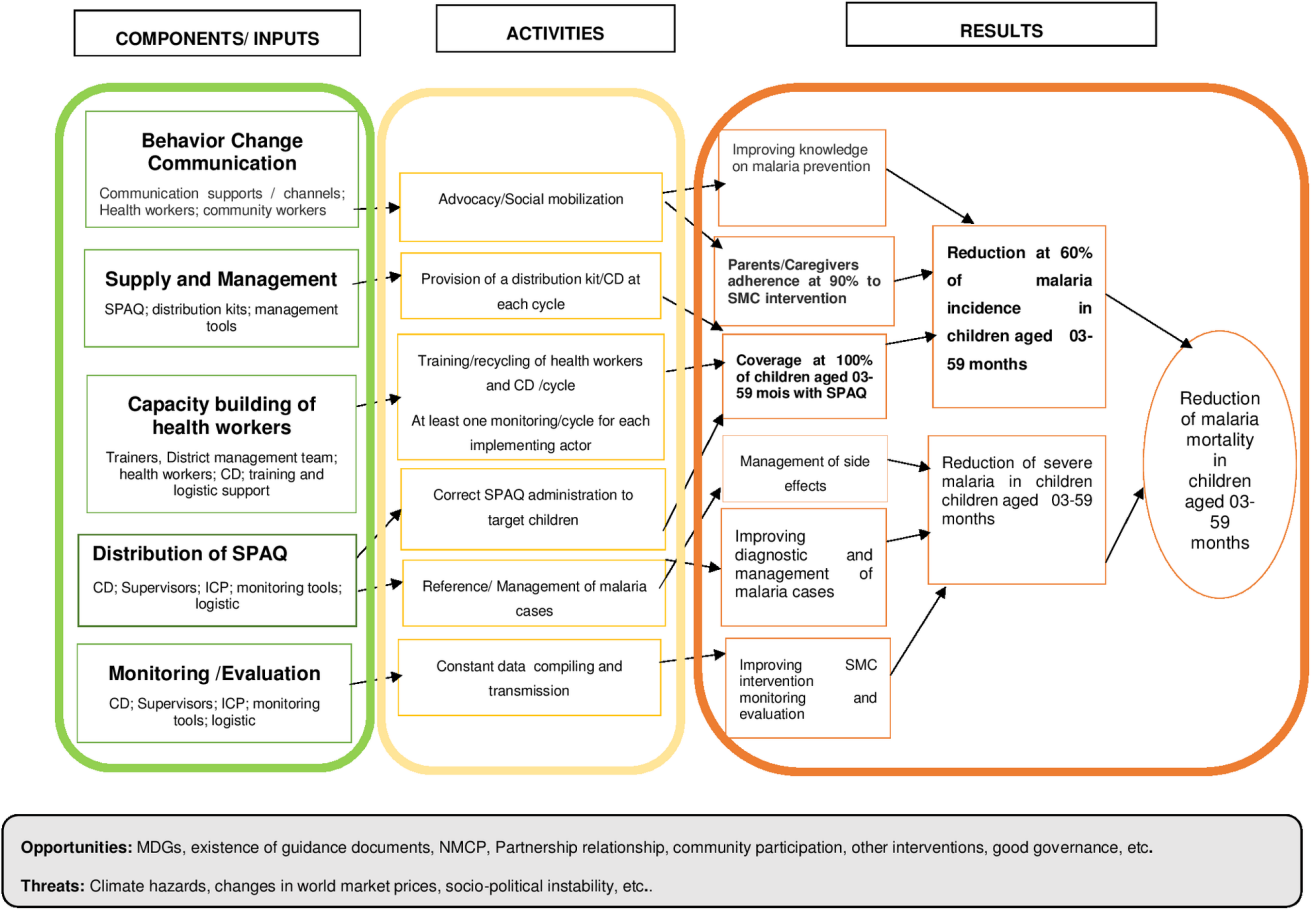
Logic model of the SMC intervention.

We assessed the evaluability of the intervention by describing the intervention theory through the reconstruction of the logic model of the intervention from the strategy planning documents and by describing the essential activities of the intervention as initially planned, as shown in Fig 1.

We then assessed the fidelity of these 39 activities by comparing implementation data to baseline project plans.

Based on the logic model, we identified and validated 39 activities grouped into five key components: behaviour change communication (BCC), supply and management of resources, capacity building of the implementing actors on the strategy, distribution of AQ+SP and finally, monitoring and evaluation of activities.

### Data collection

The data were collected in May 2016 through a review of available activity reports, semi-structured in-depth interviews (IDI) with key informants, and household surveys with caregivers of children eligible for the SMC in 2015 in the health district.

Quantitative methods were used to measure the parents’ level of knowledge of the intervention and their adherence to the SMC intervention. However, the main purpose of this approach was to estimate the coverage of children 3–59 months old during the SMC distribution campaigns, as this information was needed to validate the routinely reported treatment coverage data transmitted by the district heads of health facilities for each SMC cycle. The questionnaire was administered by five previously trained and experienced field workers to parents/caregivers of children who were eligible for SMC in Kaya Health District in 2015; data were collected directly on tablets, using CSPRO software version 6. Simple random sampling was conducted using the Kaya HDSS database to establish a list of children aged 2 to 62 months in October 2015. A simple random sample was generated from this list, and the data collectors visited each included child’s caregiver at home to administer the questionnaire. The primary outcome of the quantitative component was to determine the coverage of the intervention. Anticipating an 80% level of coverage (the lower level reported in published data to date), with a type I error of 5%, we calculated a minimum sample size of 246 needed for a margin of 5%. The minimum sample size was increased to 273 to account for a 10% non-response rate.

The intervention planning and implementation documents recommended by the National Malaria Control Program (NMCP) and available activity reports, such as staff training reports and SMC cycles’ ending reports were analysed to collect data on the activities initially planned in the programme as well as information on the actual implementation of the intervention.

Qualitative methods were used to collect data on the degree of implementation of the activities according to the different respondents as well as the moderating factors influencing the implementation of the intervention **on 2014 and 2015 SMC campaigns**. Purposive sampling was used for the individual IDIs. To include a wide range of views and experiences with the implementation of the intervention in Kaya health district, five groups of key informants were selected. The first group consisted of three district management team members who were in charge of coordinating the implementation of the intervention in Kaya health district. We then selected four rural and two semi-urban health centres based on information provided by the district management teams and a review of the SMC activity reports; a contrasting choice was made based on two criteria: coverage rate and geographical accessibility. These groups of respondents were the heads of health facilities (ICPs), supervisors, CDs and child caregivers.

We used semi-structured interview guides that were tailored to each category of respondents, and data were collected using a dictaphone and parallel note-taking.

The questionnaire and interviews were administered in French or Moore, depending on the respondent’s preference.

### Data analysis

The quantitative data were collected on tablets using CS Pro 6.0 software and were analysed using STATA 14 software. Descriptive statistics including frequencies, percentages and means were used to describe the findings.

For qualitative data, all interviews were transcribed verbatim in French before analysis using QDA Miner 4 lite software. Content analysis was used to analyse qualitative data. We developed a list of codes and themes corresponding to the dimensions of implementation fidelity chosen to analyse the SMC intervention: adherence to implementation fidelity (content, coverage, and schedule) and possible moderating factors (intervention complexity, facilitation strategies, quality of delivery, participant responsiveness and context) that might explain possible changes in fidelity over time and between health centres.

#### Analysis of adherence to intervention content and schedule

We used the analytical model proposed by Perez et al. [24] and modified by Ridde in 2013 [17]. According to this model, implementation fidelity can be quantified by determining the number of activities that are implemented as intended (I), implemented or modified (I/M),modified (M), added (A) and not implemented (N) divided by the total number of activities, for each component of the strategy.

The data collected were assessed according to the analytical framework adopted to evaluate implementation fidelity [13, 22]. We first identified a comprehensive list of planned activities by reviewing administrative documents as well as interviews with select key informants in a reasoned sample of resource persons in charge of implementing the SMC intervention in Kaya health district. We then classified this list of activities according to the main components of the intervention (supply, training, drug distribution, sensitization, monitoring and evaluation). The list of activities was then submitted to and validated by the persons in charge of the intervention at the district level.

Qualitative variables were collected on the degree of adherence of the implemented activities to initial plan through a field study in which the investigators conducted semi-structured interviews with key informant IDIs who took part in the implementation of the intervention in Kaya health district. During each interview, the respondent was asked to respond to all activities included in the list of activities that had been previously validated with actors of SMC implementation in the district; they could even, if it was considered relevant, add activities that would not have been initially planned, but were implemented. Any comments were scrupulously collected by the investigators.

The variables on fidelity of implantation of the activities collected qualitatively by interviews were then classified into the following modalities: the activity was either 1) I: implemented as planned, (2) M: modified, (3) (I/M) implemented or modified, (4) A: added (A), or (5) N: not implemented. If the respondent could remember that the activity was implemented but not how faithful it was, then it was classified as I/M (implemented or modified).

A proportion of activities classified by the respondents into the different modalities was then determined for each component of the strategy [17].

The results were then presented in the form of graphs, as proposed by Perez et al [24].

To further illustrate the quantitative proportions of content and schedule fidelity, we used the qualitative data provided by key informants and reports. We focused on activities for which dates and time periods had been specified in the planning documents to analyse adherence to the schedule.

To increase the validity, the data were reviewed and coded by two researchers for congruence.

#### Coverage data analysis

Coverage was analysed according to the process indicators proposed in the WHO field guide [3], although the indicators were adapted to our context.

The WHO SMC field guide defines coverage as the number of people reached by a programme’s services. Accordingly, we considered the coverage rates of people targeted by the national programme, namely, adherence to the intervention strategy by parents and % of eligible children treated.

For children, we used the definition proposed by the WHO, i.e., “the proportion of children who received the first dose of each treatment cycle during the transmission season”. This proportion was calculated per treatment cycle, with the number of children aged 3–59 months who received the first dose of AQ+SP as the numerator and the expected number of children aged 3–59 months in the locality during the current transmission season as the denominator. The coverage rates of courses 1, 2 and 3 (or 4, as in our context) then determined SMC coverage.

We furthered our analysis by determining the total number of doses received by each child under DOT and the total number of DOT doses each child should receive according to age group.

#### Moderating factors

Regarding moderating factors, the interviews and reports were analysed according to the themes retained in our conceptual framework.

### Ethical considerations

Written informed consent was sought from the various respondents before the data were collected.

The study was approved by the Ethics Committee for Research in Health of Burkina Faso (N° 2016-5-066).

## Results

A total of 284 parents or caregivers of children aged 0 to 62 months participated in the quantitative survey in October 2015. Children’s mothers were the most common type of respondent encountered (98%).

Twenty-one interviews were conducted to obtain the qualitative data. We interviewed three district management team members who played a key role in managing the different SMC cycles in Kaya health district, six formal health workers and six CDs from both rural and urban health facilities in the health district, and six caregivers of children eligible for the SMC intervention. Of the caregivers, only mothers participated in the interview, as it appeared that they were the primary persons caring for eligible children under five years of age.

### Implementation fidelity: Content adherence

**Fig 2:**
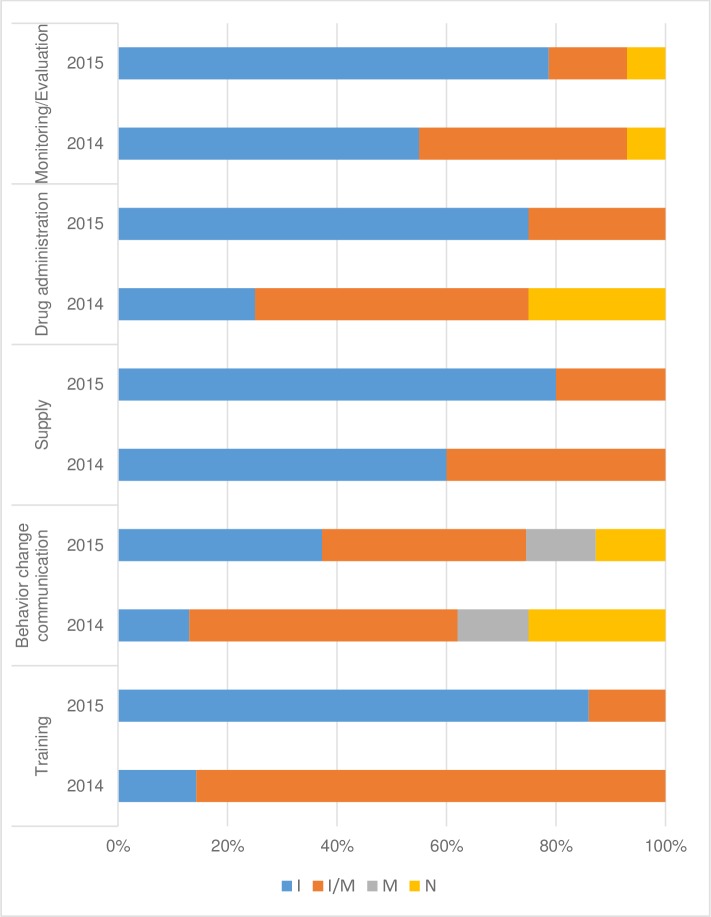
Content fidelity of the SMC components in 2014 and 2015. Notes: I = implemented as intended; I/M = implemented or modified; M = modified; N = not implemented.

Overall, all components of the SMC strategy were implemented throughout Kaya health district. The degree of adherence of the SMC intervention activities to the original intervention activities is shown in Fig 2.

During the campaigns of the two years considered in this study, no additional activities were implemented during the SMC intervention in Kaya health district. However, some activities were not implemented; these included the census of the target population of children in 2014 and 2015, as well as the distribution cycle in August 2014, because of the delay in the start of the campaign that year.

#### Staff training

Concerning the training components, in 2014, only a preparatory meeting was organized with all ICPs at all CSPSs; the ICPs, in turn, described the strategy to community volunteers at the CSPS level. One member of the Kaya district management team described this process as follows: *“people were not trained; only an information session of one day was held*.*”* However, in 2015, a three-day training of the different implementing actors in the health district was conducted. However, all preparatory meetings prior to each cycle were conducted in both 2014 and 2015.

#### Procurement and supply chain management

In terms of supply, the same difficulties, including delays and lack of certain management tools, were encountered during the 2014 and 2015 campaigns, although to a much lesser degree in 2015. As a specific example, there were no SMC administration cards during the 2014 campaign; in 2015, however, only a few parents (7.4%) had not received the SMC card when their child was given the SMC medications.

#### SMC drug administration

The instructions for administering medicines were not always followed, especially regarding the provision of the first dose by DOT: as one ICP said, *“there are CDs who agreed to give the drug to the mother for the first dose administration*… *yet*, *the first dose must be supervised when taken*.” Additionally, in 2014, some health workers participated in the campaign as CDs due to the insufficient number of qualified community volunteers.

#### Advocacy for community and social mobilization and behavioural change communication

Some difficulties were reported with the outreach component. Specifically, there were remaining gaps concerning the advocacy activities targeting local leaders, as well as parents’ sensitization during the door-to-door drug distribution. In fact, only 10% of the parents reported having been informed about the possible side effects of the treatment. Furthermore, the campaign communication material was also considered insufficient or inadequate.

#### Monitoring and evaluation

Regarding the monitoring and evaluation component, opinions varied as to the quality of the completion of these tools. One member of the district management team reported that *“in 2014*, *there were no tools to be filled out*. *By 2015*, *there were registers that the community distributors had to fill out*. *I confirm that it was not easy at all*.*”* However, data were regularly transmitted by ICPs at the end of the day throughout the cycle to the district data managers. The greatest difficulty was completing the household surveys on the days following DOT administration; for instance, an ICP stated, *“Designing an activity specifically to check if the remaining doses are given has not been done*.”

Almost all the interviewed ICPs found that the side effects of the treatment were generally under-reported. According to an ICP in a rural CSPS, *“side effects were underreported*… *it is difficult to document side effects that occurred after the team left if the parents do not present in health facilities*.*”*

However, no cases of major side effects were reported in the records of the various SMC cycles in Kaya health district.

### Implementation fidelity: Schedule adherence

**Fig 3:**
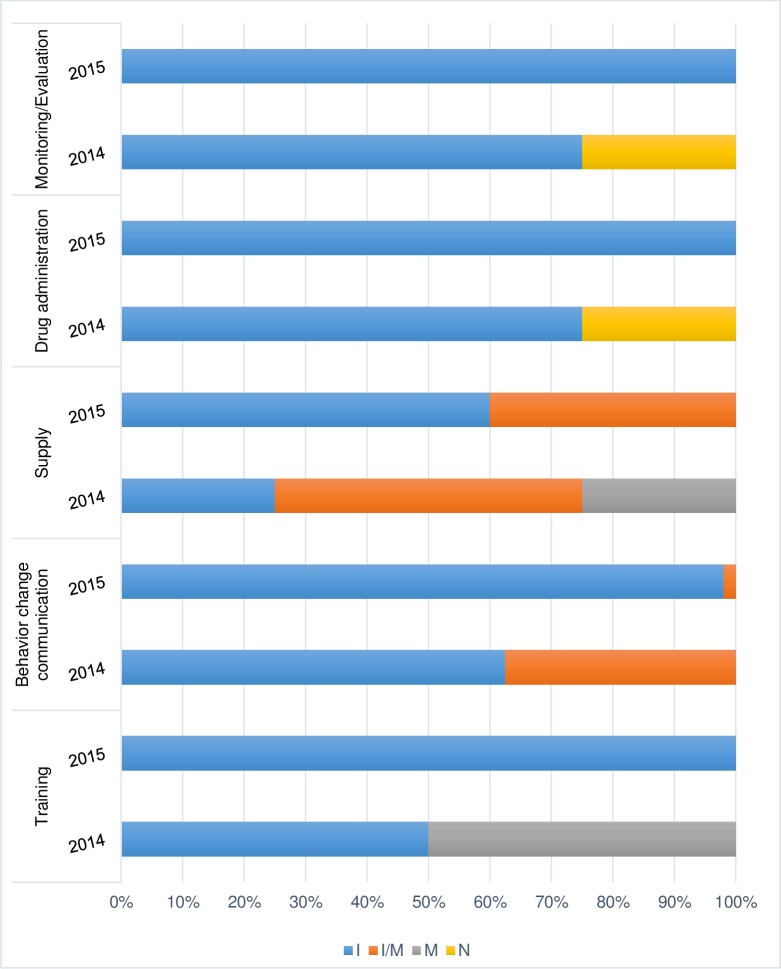
Fidelity of schedule of the SMC components in 2014 and 2015. Notes: I = implemented as intended; I/M = implemented or modified; M = modified; N = not implemented.

The adherence of the SMC activities to the initial scheduled plan was lower in 2014 than in 2015, as presented in Fig 3.

Due to the delay in starting the first SMC cycle in 2014, the campaign was shortened to three cycles instead of the four required by the SMC implementation strategy in Burkina Faso.

This delay in implementation also resulted in changes to the implementation schedule of activities such as the ICPS and CD training and advocacy among community leaders.

In addition, health facility supplies were delivered late; as one ICP reported, *“it was only the day before starting the distribution*, *in the night*, *that we received the medicines and the distribution kits*.*”*

On the other hand, there were very few changes to the implementation schedule in the 2015 SMC campaign, with an effective campaign start in late July. All four cycles proposed in the SMC implementation plan were completed on the indicated dates.

### Implementation fidelity: Coverage

The strategy was implemented in all 54 health facilities of the district from the first cycle of the 2014 campaign; all distribution cycles involved all villages and areas in the health district. As a result, 100% coverage of villages and sectors was achieved through the SMC strategy in Kaya health district in 2014 and 2015.

Almost all the parents (99.7%) provided consent for their child to receive AQ+SP, and the medication was administered at home in 96% of the cases; nearly all parents reported that they administered the remaining two (2) doses of AQ in the two days following the first administration DOT. However, due to under-reporting or low availability of the SMC cards, these data could not be cross-verified.

To evaluate the therapeutic coverage, we considered the data reported on the SMC administration cards and parents’ reports regarding the DOT administration at home. This assessment of therapeutic coverage included only the 2015 distribution campaign, as the SMC cards were not available in 2014.

Our survey showed that the SMC administration card was available in 44.7% of the cases and that approximately 7.4% of parents reported that they had not received a card during the last campaign.

#### Proportion of children who received the first dose of each treatment cycle during the transmission season

**Fig 4:**
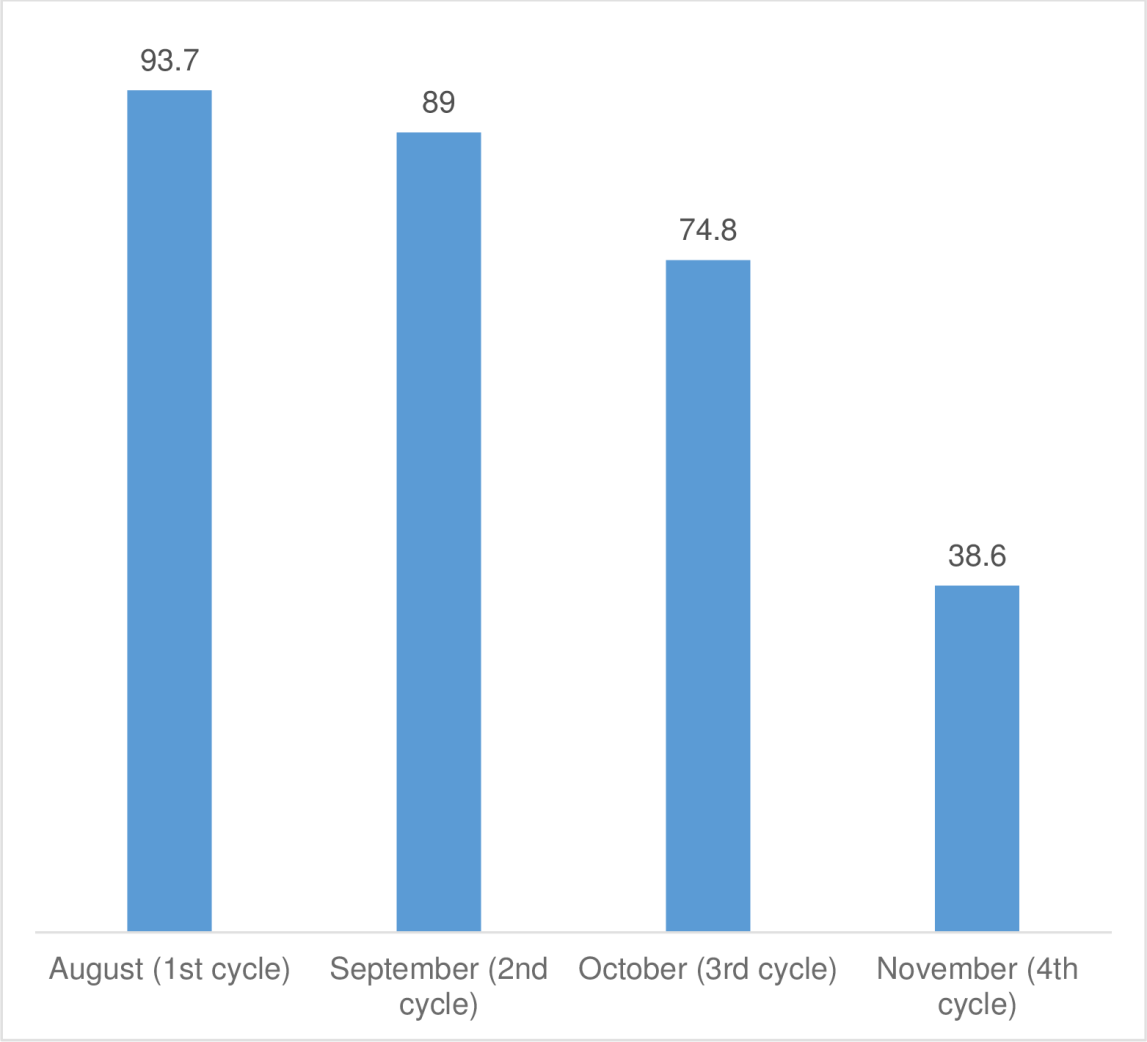
Coverage (%) per cycle of the target children with SMC DOT doses during the 2015 campaign in the Kaya health district.

According to the household survey data presented in Fig 4, the **actual coverage** of the targeted children per cycle in the four cycles was less than 100% for each cycle.

The percentage of coverage also decreased from the first to the fourth cycle in 2015, with a coverage rate of only 38.6% for the fourth cycle.

#### Proportion of children who received four complete courses during the transmission season

**Fig 5:**
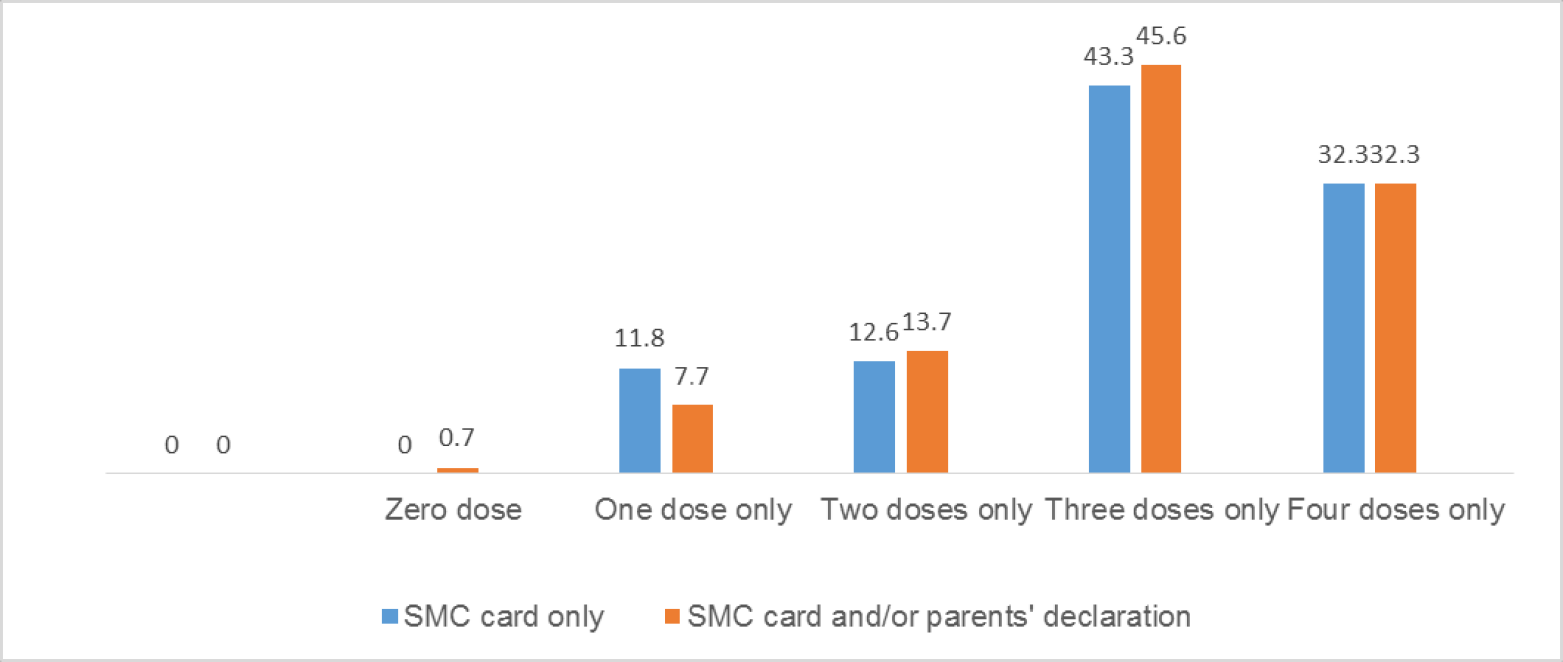
Total number of DOT doses received by the SMC target population during the 2015 campaign in Kaya health district.

The results also showed that eligible children did not receive the recommended number of doses. We found that in the sample of parents who were able to present an SMC card, approximately 75.6% of targeted children received at least three doses; of those, only 32.3% received the full course of treatment, as presented in Fig 5. We then combined the information available on the administration cards and the parents' reports and compared it with the data available on the cards only, as presented in Fig 5. Approximately 0.7% of the parents confirmed that their child had not received any SMC drugs under DOT during the 2015 campaign. The coverage of the two groups for the recommended four DOT doses was quite similar, that is, only 32.3% of eligible children received a treatment dose at each SMC cycle under DOT by CDs.

The data in Table 1 show that the eligible age for SMC administration was not always respected. Targeted children were grouped according to the number of DOT doses that each child should receive during the 2015 campaign based on their age. In Table 1, children were divided into three age groups according to the number of SMC DOT doses that each child should receive during the 2015 campaign based on their age. Thus, the group of children aged two months or less in October 2015 represented children who were 3 months old at the last cycle and should therefore have received a single DOT dose because they were eligible only for the last SMC cycle. In this group, all eight children received more doses than they should. This finding also applied for the second group of children who received more than two DOT doses. In addition, in the last group, 63% of 269 children were incorrectly treated in 2015 and did not receive the recommended four doses according to age. The eligible age of 3–59 months was therefore not always respected during the implementation of SMC in Kaya health district, and children were not always correctly treated.

**Table 1:** Percentage of children in a given age group treated with the wrong number of DOT doses during the 2015 campaign, according to the SMC card and parental report.

	Sample size	% of children incorrectly treated with the wrong DOT doses
Age group		
**[0–2]**	8	100%
**] 2–4]**	7	83%
**] 4–62]**	269	69%
**Total**	284	70%

### Moderating factors

#### Intervention complexity

In general, the main actors responsible for implementing the SMC strategy in Kaya health district agreed that the strategy was complex because of the galenic form of the drugs to administer, the tools to be completed and the large number of children to cover. For example, a CD stated the following: *“For each child*, *while one (CD) writes*, *the other will crush (the drug) with a pestle*, *mix it*, *and then wait and check if the child is vomiting*. *Then*, *you wash your utensils*, *and you have to restart all the process again for the next child*!*”*

#### Facilitation strategies

Factors such as training, supervision and the existence of field guides as well as the involvement of community leaders facilitated the implementation of the SMC strategy in Kaya health district.

#### Quality of delivery

According to the reports from most of the actors involved in implementing the SMC intervention in Kaya health district, the CDs who were already acting as community health workers (CHWs) in the home-based treatment of children with fever were more responsive to the instructions of the present intervention. Hence, the experience of being a CHW involved in malaria management in the community could constitute a quality factor in maintaining fidelity during the implementation.

#### Participant responsiveness

One of the most important moderating factors in SMC was the high level of enthusiasm for SMC; as one parent of an eligible child stated, *“It's free*, *it's effective*, *and more*, *they deliver it to our house*.*”*

In addition to these moderating factors, we also found contextual factors that affected the implementation of the SMC intervention.

#### Context

Some factors related to the context of implementation had an important influence on fidelity and, on the whole, made it difficult to implement the strategy. One of the main factors was the constraints of the rainy season. Some areas were inaccessible during the rainy season, making activities such as distribution, supervision and supply provision very difficult. As one ICP at a rural CSPS reported regarding the difficulty of access during the rainy season, “*the shallows that you have just crossed*, *when it rains*, *you cannot even cross it by car; if you are riding a bike*, *you have to drop it off and try to pass*.” Moreover, as the majority of inhabitants in Kaya health district are farmers, the parents of targeted children can be difficult to reach through the door-to-door strategy, as they leave early or may even need to move for their employment. This difficulty was explained by one CD as follows: "*This is a strategic period; if you do not leave early*, *you do not find the families in the concessions*, *you must join them in fields or farming settlements*.”

Another important factor was the lack of financial and human resources, which caused numerous complaints. For example, one member of the district management team said the following: “*In 2015*, *it was only when we realized that we were not going to have the registers that we started to make photocopies on the district’s own funds*, *and consequently*, *the tools arrived late in the CSPSs*.” An ICP also asserted, "*Only fuel is reimbursed for supervision*. *I usually take a risk; I cross the river in a flood by canoe*! *Sometimes it's 3000 XOF (~ 6 USD) or even 4500 XOF (~9 USD) to cross*, *but that is not refunded*!…*With the number of CD teams that we have*, *we are struggling to cover the sector over the four days of the cycle*.”

Other factors were also reported, such as low parental awareness of the side effects of the SMC drugs, low educational level of the population and finally, low involvement of fathers in the strategy.

In contrast to these contextual factors that negatively influenced the implementation, the health system organization, and specifically the commitment of the management team and health providers, greatly contributed to the compliance of the intervention to the national directives.

## Discussion

The objective of this study was to evaluate the fidelity of the implementation of the SMC strategy in Kaya health district using the implementation fidelity framework developed by Carroll and modified by Hasson [13, 22]. This assessment was relevant considering the need for existing programmes to study adherence to the designed intervention in order to determine whether the intervention was implemented as intended; understanding this level of fidelity is necessary before the observed effects can be attributed to the intervention and before its effectiveness can be confirmed [14,25].

Most of the components of the intervention were implemented, and all villages and sectors of the Kaya health district were covered by the intervention. However, in terms of the intervention doses received, less than a third of eligible children received the recommended four doses in 2015, and this low coverage could result in not only the failure of the intervention but also the development of antimalarial drug resistance and a risk of failure with subsequent therapy.

### Adherence of content and schedule to the initial plan

The results showed varying degrees of fidelity of the implemented activity content and schedule to the initial plan. The different levels of adherence were more apparent between the 2014 and 2015 campaigns than between the various frontline health centres in Kaya health district.

Overall, we found a better adherence of content and schedule to the planned SMC strategy in 2015 than in 2014. Durlak and DuPre in 2008 showed that perfect implementation is unrealistic and that few studies have achieved implementation levels above 80% [18]. Despite progress in recent years in measuring the quality of implementation, there is no consensus regarding how to define or measure implementation strength. According to Berman and McLaughlin, programme implementation is considered accurate if the level of implementation fidelity achieved is greater than or equal to 75% [26]. Additionally, fidelity levels that are lower than 100% enable adaptation to local contexts. Some studies have indicated that some measure of adaptation is inevitable and that better implementation occurs when some programme adjustments can be made. Thus, programme adaptation should not always be considered an implementation failure, although possible contributions of this adaptation to outcomes should be assessed.

### Coverage of targeted children

The SMC strategy was found to cover a lower percentage of 3 to 59 month-old children than desired. In Gambia and Senegal, the coverage rates during the different cycles were between 74.8 and 93.7% [9, 27]. However, the low coverage observed in our study during the fourth cycle of SMC has not yet been reported [6–9, 27, 28].

Moreover, in our study, the coverage rates were much lower than those reported by the administrative reports, which are calculated from the daily data transmitted during the SMC campaign cycles. Indeed, the monthly rates in these reports were all above 100%. According to a report by Doctors without Borders (MSF) regarding its implementation of SMC in Niger, the administrative rates of coverage varied between 157% and 174%. In the same report, however, the administrative results were found to be higher than the rates of 85% to 94% observed in a cross-sectional survey conducted during the same SMC campaign [29].

These differences could be explained by the difficulty of obtaining an exhaustive estimate of the target population. In the case of Kaya health district, uncertainty remains regarding the actual number of targeted children, despite the adjustments made from the last population census [30].

In addition, we could assume that because of the enthusiasm for the SMC strategy and the CDs’ lack of adherence to treating only eligible ages, some off-target children were also treated. This assumption may also explain the early breakdown of drug stocks that occurred despite the anticipated 15% margin incorporated based on the needs assessment. In fact, this situation raises some concerns because despite the reported stock-outs, coverage rates still exceeded 100% in the administrative rates.

As coverage is essential to assessing the impact of a control strategy for a given health problem, it is important to adopt the most appropriate methods of measuring the actual target of the SMC campaigns. Accurate methodology would ensure that the adopted strategy effectively achieves the overall objective of the SMC plan in Burkina Faso, namely, to reduce the annual incidence of malaria by at least 60% among children under five in the health districts where the strategy is implemented [4].

### Moderating factors

The complexity of the SMC strategy can be considered to be one of the main moderating factors in the implementation of the intervention. Some studies have shown that the more simple and detailed a programme is, the more likely it is to be implemented faithfully [13, 14]. According to the WHO, this complexity can explain the slow implementation observed in several countries that have adopted the SMC strategy.

Although the SMC strategy was deemed complex by most implementing actors, the potential effect of this complexity is offset by the strategies established to facilitate its implementation. Indeed, the availability of tools and field guides has been reported to facilitate the implementation of a programme as intended [13, 25]. It is undoubtedly with this objective in mind that a field guide with a detailed description of the strategy and tools for training, sensitization and monitoring was developed to implement the SMC [3, 31].

The facilitation strategies mentioned above could also positively influence the third moderating factor, which is the quality of implementation. Indeed, in studies evaluating implementation fidelity, the provision of in-depth training, tools and guides to implementing actors implicitly recognizes the effort required to optimize the quality of the intervention being applied [13,25]. Similarly, ongoing supervision and feedback on the implementation of an intervention are good signs that the quality of the implementation has been prioritized [21]. In our study, there was indeed an improvement in the quality of CD performance in distributing AQ+SP during the SMC cycles in the Kaya health district due to better training, the field guides and ongoing supervision.

Another key moderating factor in the implementation of SMC in Kaya health district was the participants’ response to the strategy. The enthusiasm of both the implementing actors and the parents of eligible children was mainly due to the perception of the effectiveness of the strategy. Thus, while Rogers proposed that preventive innovations tend to spread slowly because of the difficulty in quickly observing their benefits [32], an advantage of the SMC strategy is that the parents of the beneficiary children perceived early on that malaria episodes had been reduced in children who received SMC. This perception of the reduction of morbidity led to strong enthusiasm on the part of the health workers and the beneficiaries and is therefore an important driver of strong adherence and good engagement in the SMC strategy. Furthermore, the door-to-door distribution strategy has been recognized as the mode that allows the greatest coverage of the target population during SMC campaigns [27].

However, contextual obstacles were also noted in the implementation of the strategy. Indeed, the majority of studies on intermittent preventive treatment for malaria have focused on the technical aspects, while very few have considered the numerous organizational, social and cultural factors that could dramatically affect, either positively or negatively, the strategies’ results [6–9, 22, 27, 28].

In our study, we highlighted the challenges that the weakness of the health system, especially in terms of having sufficient financial resources and skilled providers, posed to the implementation of the strategy. Ridde et al. also emphasized this obstacle in their process evaluation of the implementation of a home-based malaria management programme (PECADO) in 2013, noting in particular the training and supervision of CHWs for community management of malaria [17].

We found the same difficulties related to the involvement of CHWs in malaria control strategies as those reported in previous studies from Senegal, Mali and Burkina Faso. These challenges included the financial motivation of the CHWs and their degree of involvement in the implementation of activities. Faye and Pitt also raised the problem of the acceptance of CHWs in urban areas [33, 34].

The methodology we selected allowed us to observe potential interactions between moderating factors, which may affect the fidelity of the implementation to the strategy, as reported in previous studies [13]. It is therefore necessary to identify the factors moderating implementation to best tailor the strategy to specific contexts and to achieve the highest possible degree of implementation fidelity by controlling these barriers [13,25].

## Conclusion

The evaluation of the implementation fidelity of the SMC showed that the content and schedule of the intervention activities adhered more to the intended plan in 2015 than in 2014. The analyses also enabled us to identify the moderating and contextual factors that could explain the poor coverage of target children or even the inadequacies of some activities.

Considering the potential of the SMC strategy to effectively combat malaria in children aged 3–59 months, appropriate measures should be taken to address the barriers to the SMC implementation in Kaya health district in particular as well as in other health districts when the intervention is scaled up.

## Supporting information

S1 File Toolsfrench. (DOCX)

S2 File Toolsenglish. (DOCX)
