# Supplementary material for: Evaluation of the implementation fidelity of the seasonal malaria chemoprevention intervention in Kaya health district, Burkina Faso
Source: PLoS One. 2017 Nov 29;12(11):e0187460. doi: 10.1371/journal.pone.0187460 (PMC5706718; doi:10.1371/journal.pone.0187460)
Supplement: S1 File — (DOCX) [file pone.0187460.s001.docx]

# Questionnaires et guides d’entretien

# Questionnaire adressé aux parents/gardiens d’enfants éligibles à la CPS

*Bonjour Monsieur/Madame, nous menons cette étude dans le cadre d’un master de recherche en santé publique. Nous voulons recueillir des informations sur les campagnes de CPS dans votre village/secteur. Avec votre permission nous allons vous poser quelques questions et cela durera une vingtaine de minutes. Acceptez-vous de participer ?* Oui /--/ Non /--/

*Nom de l’enquêteur……………………………………………………………………………*

*Date de l'entretien :……………………………………………………………………………*

*Village/secteur ……………………………………………..*

*Code village/secteur* /---//---/

*Bloc* /---/

*Concession* /---//---//---/

*Ménage* /---//---/

*Code*/---//---//---//---//---//---//---//---//---//---/

1. Caractéristiques socio-démographiques de l’enquêté

Q1. Sexe de l’enfant 1. Masculin /--/ 2.Feminin /--/

Q2. Quel est l’âge de votre enfant ? /---/--/ (en mois révolus)

Q3.Quel âge avez-vous? /---/--/ (en année révolues)

Q4. Sexe du parent/tuteur

1. Masculin /--/ 2. Féminin /--/

Q5. Quel est votre lien avec l’enfant

1. Père /--/ 2. Mère /--/ 3. Autre (préciser) /--/ ………………………….

Q6.Quel est votre statut matrimonial?

1. Mariée /--/ 2. Veuve /--/ 3. Célibataire /---/ 4. Divorcé /--/

Q7. Quelle est votre profession ?

1. Fonctionnaire /--/ 2. Commerçant(e) /--/ 3. Ménagère /--/ 4. Cultivateur /--/

5. Eleveur/--/ 6. Elève/Etudiant /--/ 7. Sans profession /--/ 8. Autre /--/……...

Q8.Quel est votre niveau d’instruction ?

1. Sans niveau /--/ 2. Primaire /---/ 3.Secondaire /--/ 4. Supérieur /--/

Q9.Quel est votre religion ?

1 Islam /--/ 2 Animisme /--/ 3 Christianisme/--/

Q10.Quelle est votre ethnie ?

1. Mossi /--/ 2 Peulh /--/ 3 Autres /--/
2. Appréciation de la Communication sur la CPS

Q9. Avez-vous déjà entendu parler de la CPS ?

1. Oui /--/ 2. Non /--/

Q10. Si oui, comment avez-vous obtenu l'information sur la CPS ?

1. Equipe de Distribution /--/
2. Radio = /--/
3. Télévision /--/
4. Banderole /--/
5. Agent de Santé Com. /--/
6. Chefs religieux et coutumiers /-/
7. Agents de santé /--/
8. Crieur Public /--/
9. Voisin /--/
10. Autres /--/
11. Préciser………………………

Q11. Savez-vous contre quelle maladie donne-t-on le médicament de la CPS?

| 1. Paludisme /--/ | 2. Autres Maladies /--/ | 3. Ne sait pas /--/  Si autre, préciser………………… |
| --- | --- | --- |

Q12. Avez-vous reçu des explications sur la CPS ?

1. Oui /--/ 2.Non /--/

Q13. Si oui, à quel moment ?

1. Avant le passage des distributeurs communautaires /--/
2. Au moment de la distribution /--/
3. A une autre occasion /--/

Si 3, préciser………………………………..

Q14. Sur quoi ont porté ces explications (cocher si cité)

1. Sur la stratégie CPS /--/
2. Sur les autres méthodes de prévention du paludisme /--/
3. Sur le mode d’administration des doses d’AQ, à domicile /--/
4. Sur les effets secondaires /--/

Q15. Avez-vous compris ses explications ?

1. Non/--/ 2. Oui toutes/--/ 3. Oui certaines 4.Sans opinion /--/

1. Adhésion à la CPS

Q16. Votre concession a-t-elle été visitée lors de la campagne CPS de 2015 ?

1. Oui /--/ 2. Non /--/

Q17. Si oui, combien de fois avez-vu reçu la visite des DC dans le cadre de la CPS ? /--/--/

Q18. Combien d’enfants de 03-59 mois aviez-vous sous votre responsabilité lors de la dernière campagne CPS ? /--/--/

Q19. Avez-vous donné votre accord pour l’administration des médicaments à votre enfant lors de la dernière campagne de CPS ? (si oui, aller à Q21)

1. Oui /--/ 2.Non /--/

Q20. Si non, pourquoi ne voulez-vous pas donner les comprimés à vos enfants?

1. Médicaments dangereux /--/
2. Equipe pas courtoise /--/
3. Ce n'est pas moi qui décide /--/
4. Ne sais pas /--/
5. Pas de réponse /--/
6. Autres /--/ ………………..
7. Administration de l’AQ-SP aux enfants éligibles

Q21. Votre enfant a-t-il reçu les médicaments en traitement directement observé lors du passage des distributeurs communautaires ?

1. Oui /--/ 2. Non /--/

Q22. Si oui, où est ce que l’enfant a reçu le médicament

1. A la maison /--/ 2. Au CSPS /--/ 3. Autre endroit /--/

Q23. Si non, pourquoi l’enfant n’a pas reçu le médicament

1. Absence /--/ 2. Sous traitement paludisme /--/ 3. Autre /--/…………………

Q24. Votre enfant a-t-il reçu le médicament à la maison les deux jours suivant la première administration ?

1. Oui /--/ 2.Non /--/

Q25. Si non, pourquoi ?

1. Vomissement /--/
2. Peur des effets secondaires /--/
3. Oubli /--/
4. Absence du parent /--/
5. Absence de l’enfant /--/
6. Ne savait pas comment donner le médicament /--/
7. Ne voulait pas donner le médicament /--/

Q26. Savez-vous que la dose est différente pour les enfants de 3-11 mois et 12-59 mois ?

1. Oui /--/ 2.Non /--/

Q27. Avez-vous la carte où on coche l’administration des médicaments ?

1. Oui (vu) /--/ 2. Oui (non vu)/--/ Non /--/

Si 1, préciser si médicament administré :

2015 : mois 1 /--/ mois 2 /--/ mois 3 /--/ mois 4 /--/

Q28. Etes-vous satisfait de la manière dont les médicaments sont distribués aux enfants ?

1. Très satisfait /--/ 2. Satisfait /--/ 3. Passablement satisfait 4 Pas satisfait

Q29. Si non (Si Q28 = 2,3,4), préciser :

1. Information/Communication /--/
2. Période d’administration/--/
3. Comportement des distributeurs communautaires /--/
4. Effets secondaires /--/
5. Autres /--/ …………………

Fin de l’interview

Merci pour votre participation!

# Guides d’entretien semi-directifs pour l’enquête qualitative

**Guide d’entretien individuel : Médecin chef de District**

*Bonjour Monsieur/Madame. Nous sommes étudiante à l’IASP et nous menons cette étude dans le cadre de notre master en santé publique. Nous voulons nous entretenir avec vous de la mise en œuvre de la stratégie CPS dans votre district. Acceptez-vous de participer à notre enquête ?*

*Oui /--/ 2.Non /--/*

*Nom de l’enquêteur……………………………………………………………………………*

*Date de l’enquête………………………………………………………………………………*

1. Quel est le rôle de l’ECD dans l’implantation de la CPS dans le district sanitaire ?
2. Comment se prépare les campagnes et chaque cycle de CPS au niveau de votre district ?
3. Comment se déroulent les campagnes et les cycles de CPS au niveau de votre district ?
   - Formation
   - Approvisionnement
   - Distribution
   - Supervision
   - Sensibilisation
   - Transmission des données
   - Calendrier des activités
4. Y a-t-il des activités prévues qui n’ont pas pu se tenir ? (oui, non) Si oui, citer et développer les types d’activités réalisées, les activités non réalisées et les éventuelles causes.
5. Y a-t-il des activités prévues qui ont été modifiées à la mise en œuvre ? Si oui donner les éventuelles causes.
6. Que pensez-vous du degré de mise en œuvre des activités entrant dans le cadre de ce programme ?
7. Pensez-vous que la CPS puisse être intégrée à d’autres activités dans le district? Lesquelles ?
8. La CPS est-elle intégrée à d’autres activités dans votre district ?
9. Comment impliquez-vous les communautés dans les campagnes CPS au niveau de votre district ?
10. Comment jugez-vous la participation de la communauté à la CPS?
11. Quels sont les messages sur lesquels vous insistez lors des campagnes de CPS dans votre district ?
12. Quelles sont les difficultés et les contraintes que vous rencontrez dans l’organisation et le déroulement des campagnes ?
13. Comment avez-vous géré ces difficultés ?
14. Quels sont les facteurs qui, selon vous, ont favorisé la mise en œuvre des campagnes de CPS dans votre district sanitaire (organisation et déroulement des campagnes) ?
15. Avez-vous des suggestions pour l’amélioration des campagnes de CPS dans les districts sanitaires ?

Fin de l’interview

Merci pour votre participation!

**Guide d’entretien adressé aux agents de santé dans les formations sanitaires**

*Bonjour Monsieur/Madame. Nous sommes des étudiants de l’IASP et nous menons cette étude dans le cadre de notre master en santé publique. Nous allons discuter avec vous dans le cadre de la prise en charge communautaire de la malnutrition mis en place dans votre district. Vous êtes invités à répondre aux questions et à discuter avec nous sur sa mise en œuvre.*

Nom de l’enquêteur……………………………………………………………………………

Date de l’enquête………………………………………………………………………………

Caractéristiques de l’enquêté

Sexe |___| (1) Masculin (2) Féminin

Qualification …………………………………………………………………………………..

Structure ………………………………………………………………………………………

1. Quel est votre rôle dans l’implantation de la CPS dans le district sanitaire ?
2. Comment se prépare les campagnes et chaque cycle de CPS au niveau de votre CSPS ?
3. Aviez-vous prévu une un circuit de distribution ?
4. Comment se déroulent les campagnes et les cycles de CPS au niveau de votre CSPS ?

- Formation
- Approvisionnement
- Distribution
- Supervision
- Sensibilisation
- Remplissage des formulaires et transmission des données
- Calendrier

1. . Y a-t-il des activités prévues qui n’ont pas pu se tenir ?

Si oui, citer et développer les types d’activités réalisées, les activités non réalisées et les éventuelles causes.

1. Y a-t-il des activités prévues qui ont été modifiées à la mise en œuvre ? Si oui donner les éventuelles causes.
2. Que pensez-vous du degré de mise en œuvre des activités entrant dans le cadre de ce programme ?
3. La CPS est-elle intégrée à d’autres activités dans votre district ?
4. Comment impliquez-vous les communautés dans les campagnes CPS au niveau de votre district ?
5. Comment jugez-vous participation de la communauté à la CPS?
6. Quels sont les messages sur lesquels vous insistez lors des campagnes de CPS dans votre CSPS?
7. Quelles sont les difficultés et les contraintes que vous rencontrez dans l’organisation et le déroulement des campagnes ?
8. Quels sont les facteurs qui, selon vous, ont favorisé la mise en œuvre des campagnes de CPS dans votre CSPS (organisation et déroulement des campagnes) ?
9. Avez-vous des suggestions pour l’amélioration des campagnes de CPS dans les aires sanitaires des CSPS?

Fin de l’interview

Merci pour votre participation!

**Guide d’entretien individuel : Distributeurs communautaires**

*Bonjour Messieurs/Mesdames, nous sommes des étudiants de l’IASP et nous menons cette étude dans le cadre de notre master en santé publique. Nous voulons discuter avec vous à propos de l’intervention sur la chimio prophylaxie du paludisme saisonnier (CPS) mis en place dans votre district. Vous êtes invités à répondre aux questions et à discuter avec nous sur sa mise en œuvre. Acceptez-vous de participer ?* Oui /--/ 2.Non /--/

Nom de l’enquêteur……………………………………………………………………………

Date de l'entretien :……………………………………………………………………………

Q1.Village/secteur de …………………………………………………………………

Q2.Code /___/

Caractéristiques de l’enquêté

Sexe |___| (1) Masculin (2) Féminin

Age |___|

1. Que savez-vous de la stratégie CPS?
2. Comment est-elle mise en œuvre dans votre zone sanitaire ?
3. Comment se déroulent les cycles de distribution de la CPS dans votre zone ?
4. Comment appréciez-vous la mise en œuvre des activités prévues dans le cadre de la CPS?

- Formation
- Approvisionnement
- Distribution
- Supervision
- Sensibilisation
- Remplissage des formulaires et transmission des données
- Calendrier
- Implication de la communauté

1. Quels sont les messages sur lesquels vous insistez lors de la campagne CPS ?
2. Que pensez-vous du degré de mise en œuvre des activités entrant dans le cadre de ce programme ? Y a-t-il des points à améliorer dans la mise en œuvre de ce programme ? Si oui, listez.
3. Quelles sont les difficultés que vous rencontrez dans la mise en œuvre ?
4. Qu’est-ce qui, selon vous a favorisé le déroulement des campagnes ?
5. Avez-vous des suggestions pour améliorer la mise en œuvre de ce programme ?

Fin de l’interview

Merci de nous avoir accordé de votre temps !

**Guide d’entretien individuel : leaders communautaires**

*Bonjour Monsieur/Madame, nous sommes étudiants à l’IASP et nous menons cette étude dans le cadre de notre master en santé publique. Nous voulons recueillir des informations sur les campagnes de CPS dans votre village/secteur. Avec votre permission nous allons vous poser quelques questions et cela durera une vingtaine de minutes. Acceptez-vous de participer ? Oui /--/ 2.Non /--/*

*Nom de l’enquêteur……………………………………………………………………………*

*Date de l'entretien :……………………………………………………………………………*

*Q1.Village/secteur de …………………………………………………………………*

*Q2.Code /___/*

1. Savez-vous ce qu’est la CPS ?
2. Si oui, comment en avez-vous attendu parler ?
3. Avez-vous été informé lors des campagnes de CPS ?
4. Comment avez-vous participé à la campagne CPS ?
5. Que pensez-vous du déroulement des campagnes ?
6. Quels sont les messages qui ont été transmis :
   1. Sur la CPS
   2. Sur le paludisme
7. Qu’est-ce que vous avez apprécié lors des campagnes ?
8. Qu’est-ce que vous n’avez pas apprécié ?
9. Avez-vous des recommandations/suggestions à faire pour l’amélioration du programme ?

Fin de l’interview

Merci pour votre participation!

**Guide d’entretien individuel : Parent/Gardien d’enfant**

*Bonjour Monsieur/Madame, nous sommes étudiants à l’IASP et nous menons cette étude dans le cadre de notre master en santé publique. Nous voulons recueillir des informations sur les campagnes de CPS dans votre village/secteur. Avec votre permission nous allons vous poser quelques questions et cela durera une vingtaine de minutes. Acceptez-vous de participer ? Oui /--/ 2.Non /--/*

*Nom de l’enquêteur……………………………………………………………………………*

*Date de l'entretien :……………………………………………………………………………*

*Q1.Village/secteur de …………………………………………………………………*

*Q2.Code /___/*

1. Savez-vous ce qu’est la CPS ?
2. Si oui, comment en avez-vous attendu parler ?
3. Avez-vous accepté que votre enfant prenne le médicament ?
4. Vous –a-t-on montré comment vous devez donner le médicament à votre enfant les deux jours qui suivent l’administration par l’ASC?
5. Avez-vous administré les deux doses de médicaments les deux jours qui ont suivi la première prise données par les agents de santé communautaires ?
6. Comment trouvez-vous l’administration du médicament ?
7. Avez-vous expérimenté des effets indésirables ?
8. Si oui, qu’avez-vous fait ?
9. Lors des campagnes, vous –a-t-on conseillé sur comment se protéger contre le paludisme ?
10. Si oui, que vous a-t-on dit?
11. Qu’est-ce que vous avez apprécié lors des campagnes ?
12. Qu’est-ce que vous n’avez pas apprécié ?
13. Avez-vous des recommandations/suggestions à faire pour l’amélioration du programme ?

Fin de l’interview

Merci pour votre participation!

# Formulaire d’information et de consentement éclairé

**INFORMATION**

**Introduction**

Bonjour, Madame/Monsieur, je m’appelle COMPAORE RACHIDATOU et je suis étudiante à l’institut africain de santé publique (IASP). Nous sommes ici pour réaliser une étude dans le cadre de notre mémoire pour l’obtention d’un master 2 en santé publique option planification et gestion des services de santé. Cette étude s’intitule : « évaluation du processus de mise en œuvre de la stratégie CPS dans le district sanitaire de Kaya ».

J’enquête donc sur la mise en œuvre de la chimio-prévention du paludisme saisonnier dans le district sanitaire de Kaya. Voulez-vous en apprendre plus sur cette enquête en vue d’y participer ? (Enquêteur : si ‘oui’, continuer. Si ‘non’, remercier et terminer l’entretien.

**But de la recherche**

Le paludisme reste un véritable problème de santé publique au Burkina, surtout pour les enfants de moins de cinq (05) ans chez lesquels il représente la première de cause de morbidité et de mortalité. Cette étude se propose d’évaluer la mise en œuvre de la CPS, qui a été reconnue comme une stratégie efficace de lutte contre le paludisme chez les enfants de 03-59 mois. Nous espérons que les résultats de cette étude apporteront des informations au public ainsi qu’aux décideurs de la santé et aux gestionnaires de programmes afin qu'ils puissent étendre et assurer la durabilité de ces interventions appropriées pour réduire la charge de morbi-mortalité due au paludisme.

**Sélection des participants**

Vous êtes invité à participer à cette recherche parce que nous pensons que l’expérience de campagnes de distribution que vous avez vécue peut contribuer beaucoup à améliorer le processus de mise en œuvre d’autres programmes de nutrition.

**Participation volontaire**

Votre participation à cette étude est entièrement volontaire. Vous pouvez arrêter et vous retirer de l'entretien à n'importe quel moment sans aucun effet sur les services de santé dont vous ou un membre de votre famille pourriez avoir besoin dans l'avenir. Sentez-vous libre de me le faire savoir si vous n’êtes plus disposé à continuer cet entretien.

**Procédures**

Nous vous invitons donc à prendre part à un entretien qui va durer à peu près 20-30 minutes de votre temps. Cet entretien va porter sur les l’organisation des campagnes, la sensibilisation sur la CPS et la distribution des médicaments. Nous allons vous poser des questions et tout ce que vous pourrez dire sur le sujet nous intéresse.

**Confidentialité**

L’information que vous partagez avec nous sera gardée confidentielle; elle ne sera utilisée que pour l’étude. Le mémoire utilisera les réponses collectives et ne révélera ni les noms ni aucun indice qui pourrait être relié à la personne qui aura donné l’information. Aucune personne non plus qui n’est pas directement impliquée dans cette étude ne sera autorisée à accéder aux informations que vous nous donnez. Si tous les résultats de cette étude sont édités, votre identité demeurera confidentielle.

Mais il ne sera nullement fait mention de votre identité ou de toute déclaration susceptible de vous reconnaître. Les informations divulguées seront totalement anonymes.

Ce formulaire de consentement qui comporte votre nom sera tenu séparé du questionnaire et sera détruit dans une année.

**Risques et avantages**

La participation à l’étude n’apporte pas une compensation en nature pour chacun des participants, mais les connaissances que vous nous permettrez d’acquérir peuvent contribuer à prendre des mesures pour améliorer la santé des enfants de moins de 05 ans.

Nous allons préserver la confidentialité des informations, mais ne pouvons pas garantir toute violation qui pourrait arriver. Cependant, les questionnaires et les fichiers ne contiennent aucune information d'identification personnelle et, par conséquent, votre participation et informations resteront confidentielles.

**Contact**

Ce projet est mené par l’Institut Africain de Santé Publique (IASP). Si vous avez besoin de contacter ce projet de recherche à une date ultérieure, vous pouvez communiquer avec COMPAORE Rachidatou au numéro 72 37 56 12.

Si l'information que je vous donne n’est pas claire ou si vous avez des questions au sujet de cette recherche et cette interview, vous pouvez me demander maintenant. Avez-vous des questions à me poser?

**CERTIFICAT DE CONSENTEMENT ECLAIRE**

J’ai été informé sur cette recherche qui porte sur l'évaluation de la mise en œuvre de la CPS dans le district sanitaire de Kaya. J'ai lu le formulaire de consentement éclairé ou il m’a été lu; J'ai eu l'occasion de poser des questions sur la recherche et mes questions ont été clairement expliquées à ma satisfaction.

Je comprends et je consens à participer à étude et à permettre l’utilisation des transcriptions faites dans le cadre de cette étude : Oui /--/ Non /--/

Si oui, ma signature ou mon empreinte digitale ci-dessous indique que je comprends le but, les risques et les avantages de cette étude et que j’ai eu des réponses à mes questions. On m’a fourni les coordonnées de celui que je peux contacter si j’ai des questions ou des plaintes. Par conséquent, je décide de participer volontairement aux échanges entrant dans le cadre de cette étude.

**Signature ou empreinte digitale** ……………………………………………………………..

**Date**: ……/……../…….
