# Supplementary material for: Evaluation of the implementation fidelity of the seasonal malaria chemoprevention intervention in Kaya health district, Burkina Faso
Source: PLoS One. 2017 Nov 29;12(11):e0187460. doi: 10.1371/journal.pone.0187460 (PMC5706718; doi:10.1371/journal.pone.0187460)
Supplement: S2 File — (DOCX) [file pone.0187460.s002.docx]

**Questionnaires and interview guides**

**Questionnaire to parents / caregivers’ of children eligible for SMC**

*Dear Sir / Madam, we are conducting this study as part of a Master's in Public Health Research.* *We want to collect information about SMC campaigns in your village / area.* *With your permission we will ask you a few questions ; this could last about twenty minutes.* *Do you agree to participate?* Yes/--/ No /--/

*Investigator’s name .......................................................................................*

*Date of interview: .......................................................................................*

*Village / area ................................................... ..*

*Code Village / sector* / --- // --- /

*Block* / --- /+-+

*Concession* / --- // --- // --- /

*Householdjmjl* / --- // --- /

*Code* / --- --- // // // --- --- --- // // // --- --- --- // // // --- --- /

1. Socio-demographic characteristics

Q1. Sex of the child 1. Male / - / 2.Feminin / - /

Q2. How old is your child? / --- / - / (in completed months)

Q3. How old are you? / --- / - / (in completed years)

Q4. Parent / caregiver’s sex

1. Male / - /  2. Female / - /

Q5. What is your relationship with the child

1. Father / - / 2. Mother / - / 3. Other (precise) / - / ...............................

Q6.What is your marital status?

1. Maried / - / 2. Widow / - / 3. Single / --- / 4.   Divorced / - /

Q7. What is your profession ?

1. Officer / - / 2. Trader / - /  3. Housewife / - / 4. Farmer / - /

5. livestock breeder / - / 6. Student / - / 7. No occupation / - / 8. Other / - / ...... ...

Q8.What is your level of education?

1. Not educated / - / 2. Primary / --- / 3.Secondary / - / 4. Tertiary / - /

Q9.What is your religion?

1 Muslim / - / 2 Animist / - / 3 Christian / - /

Q10. What is your ethnic group?

1. Mossi / - / 2 Peulh / - / 3 Others / - /
2. Opinion on SMC  Communication strategy

Q9. Have you ever heard of SMC?

1. Yes / - / 2. No / - /

Q10. If yes, how have you heard about SMC?

1. Distribution Team / - /
2. Radio = / - /
3. Television / - /
4. Banner / - /
5. Community health worker. / - /
6. Religious and/or traditional leaders / - /
7. Formal health worker / - /
8. Town crier / - /
9. Neighbour /--/
10. Other / - /

Specify ...........................

Q11. Do you know against which disease are the SMC drugs fighting against ?

| 1. Malaria / - / | 2. Other Diseases / - /  If other, specify………………… | 3. Do not know / - / |  |
| --- | --- | --- | --- |

Q12. Did you receive any explanation about SMC?

1. Yes / - / 2.No / - /

Q13. If so, when?

1. Before the passage of community distributors / - /
2. At the time of distribution / - /
3. On another occasion / - /

If 3, specify .................................... ..

Q14. What were these explanations on ? (tick if so quoted)

1. On SMC strategy / - /
2. On the other methods of malaria prevention / - /
3. On the mode of administration of doses of AQ at  home by parents / - /
4. On the drugs’ side effects / - /

Q15. Did you understand these explanations?

1. No / - / 2. Yes all of them / - / 3. Yes some of them 4.No opinion / - /

1. Adhesion to SMC strategy

Q16. Was your house visited during the 2015 SMC campaign?

1. Yes / - / 2. No / - /

Q17. If so, how many times did community distributors visited your house last year? / - / - /

Q18. How many children aged 3 to 59 months where under your responsibility during the last SMC campaign? / - / - /

Q19. Have you given your consent for the drugs’ administration to your child during the last SMC campaign? (If yes, go to Q21)

1. Yes / - / 2.No / - /

Q20. If not, why ?

1. Because the drugs are dangerous / - /
2. The team was not courteous / - /
3. It's not me who decides / - /
4. Do not know /--/
5. No response  / - /

Other / - / .................. ..

1. Administration of SP-AQ to eligible children

Q21. Was the drugs administered to your child received in directly observed treatment by community distributors?

1. Yes / - / 2. No /--/

Q22. If yes, where did the child receive the drug ?

1. At home / - / 2. At the health facility / - / 3. Other location / - /

Q23. If not, why didn’t the child receive the medication ?

1. was absent / - / 2. was under malaria treatment / - / 3. Other / - / .....................

Q24. Did your child receive the rest of the medication at home two days after the first administration?

- 1. Yes / - / 2.No / - /

Q25. If not why ?

1. Vomiting / - /
2. Fear of side effects / - /
3. The caregiver’s has forgotten the dose / - /
4. Absence of the caregiver / - /
5. Absence of the child / - /
6. Did not how to give the drug/ - /
7. Did not want to give the drug to the child / - /

Q26. Do you know that the dose is different between children of 3-11 months and 12-59 months?

1. Yes / - / 2.No / - /

Q27. Do you have the card on which SMC drug administration is checked?

1. Yes (seen) / - / 2. Yes (not seen) / - / No / - /

If yes, specify if drug was administered in:

2015: month 1 / - / month 2 / - / month 3 / - / month 4 / - /

Q28. Are you satisfied with the way the drugs are distributed to children?

- 1. Very satisfied / - / 2. Satisfied / - / 3. Fairly satisfied / - / 4 Not satisfied/ - /

Q29. If not satisfied  (if Q28 = 3,4), please precise on which aspect:

1. Information / Communication / - /
2. Drug administration period / - /
3. Behavior of Community distributors / - /
4. Side effects /--/
5. Other / - /        .....................

End of interview

Thank you for your participation!

**Individual interview guides**

**Individual interview guide with the District Chief Medical Officer**

*Dear sir/madam, we are conducting this study as part of our Master's in Public Health.* *We want to talk to you about the implementation of the SMC strategy in your district.* *Do you agree to participate in our survey?*

*Yes / - / 2.No / - /*

*Name of Investigator .......................................................................................*

*Date of investigation ..........................................................................................*

1. What is your role  in the implementation of SMC in the health district?
2. How do you plan each SMC campaign and each SMC cycle in your district?
3. How do SMC cycles usually go on in your district?
   - Training
   - Procurement
   - Distribution
   - Supervision
   - Sensitization
   - Data transmission
   - Activity planning
4. Is there any planned activity which could not been implemented? (yes, no) If yes, report it and develop the types of activities performed, not performed, and possible causes.

Are there any planned activities that have been modified at the implementation? If yes, give the possible causes.

1. What do you think of the degree of implementation of the activities covered by this program?
2. Please quote the degree to which each activity as been implemented as initially planned.
3. Do you think that SMC can be integrated into other activities in the district? Which ones?
4. Is SMC integrated with other activities in your district?
5. How do you involve communities in SMC campaigns at your district level?
6. How do you assess participation of the community at SMC campaigns?
7. What are the messages of sensitizationon which you insist during SMC in the health district?
8. What are the difficulties and constraints you face in implementing SMC campaigns?
9. What are the factors that you think may favor SMC intervention implementation in the health district?
10. Do you have suggestions to improve SMC implementation of SMC in the health district?

End of interview

Thank you for your participation!

**Interview guide with formal health workers**

*Dear sir/madam, we are conducting this study as part of our Master's in Public Health.* *We want to talk to you about the implementation of the SMC strategy in your district.* *Do you agree to participate in our survey*

Name of Investigator .......................................................................................

Date of investigation ..........................................................................................

Characteristics of respondent

Sex | ___ | (1) Male (2) Female

Qualification ............................................................................................. ..

Structure ...................................................................................................

1. What is your role  in the implementation of SMC in the health district?
2. How is planned each SMC campaign and each SMC cycle in your district?
3. How do SMC cycles usually go on in your district?
   1. Training
   2. procurement
   3. Distribution
   4. supervision
   5. Sensitization
   6. Data transmission
   7. Activity planning
4. Is there any planned distribution circuit for SMC cycles?
5. Is there any planned activity which could not been implemented? (yes, no) If yes, report it and develop the types of activities performed, not performed, and possible causes.
6. Are there any planned activities that have been modified at the implementation? If yes, give the possible causes.
7. What do you think of the degree of implementation of the activities covered by this program?
8. Please quote the degree to which each activity as been implemented as initially planned.
9. Do you think that SMC can be integrated into other activities in the district? Which ones?
10. Is SMC integrated with other activities in your district?
11. How do you involve communities in SMC campaigns at your district level?
12. How do you involve the community in SMC campaigns in your district?
13. How do you assess participation of the community at  SMC campaigns?
14. What are the messages of sensitizationon which you insist during SMC in the health district?
15. What are the difficulties  and constraints you face in implementing SMC campaigns?
16. What are the factors that you think may favor SMC intervention implementation in the health district?
17. Do you have suggestions to improve SMC implementation of SMC in the health district?

End of interview

Thank you for your participation!

**Individual interview guide with Community Distributors**

*Dear sir/madam, we are conducting this study as part of our Master's in Public Health.* *We want to talk to you about the implementation of the SMC strategy in your district.* *Do you agree to participate in our survey*

Yes / - / 2.No / - /

Name of Investigator .......................................................................................

Date of interview: .......................................................................................

Q1.Village / area of ​​...........................................................................

Q2.Code / ___ /

Characteristics of the respondent

Sex | ___ | (1) Male (2) Female

Age | ___ |

- 1. What do you know of the SMC intervention?
  2. How is it carried out in your health area? How do SMC cycles usually go on in your district?
     1. Training
     2. procurement
     3. Distribution
     4. supervision
     5. Sensitization
     6. Data transmission
     7. Activity planning
  3. What messages are you insisting on during the SMC campaign?
  4. What do you think of the degree of implementation of the activities covered by this program?
  5. Please quote the degree to which each activity as been implemented as initially planned.
  6. Are there any areas for improvement in the implementation of this program? If so, list.
  7. What are the difficulties you encounter in the implementation of the SMC intervention?
  8. What do you think has favored the implementation of the campaign?
  9. Do you have any suggestions for improving the implementation of this program?

End of interview

Thank you for your time!

**Individual Interview Guide with community leaders**

*Dear sir/madam, we are conducting this study as part of our Master's in Public Health.* *We want to talk to you about the implementation of the SMC strategy in your area.* *Do you agree to participate in our survey*

Yes / - / 2.No / - /

*Name of Investigator .......................................................................................*

*Date of interview: .......................................................................................*

*Q1.Village / area of ​​...........................................................................*

*Q2.Code / ___ /*

1. Do you know what  SMC is?
2. If so, how did you heard about it?
3. Have you been informed of the SMC campaign?
4. How did you take part in SMC campaign?
5. What do you think of the SMC campaigns implementation?
6. What are the messages that have been passed :
   1. about SMC
   2. About malaria
7. What did you like during the campaigns?
8. What didn’t you appreciate?
9. Do you have any recommendations / suggestions for improving the program?

End of interview

Thank you for your participation!

**Individual interview guide with Parents / Caregivers**

*Dear Sir / Madam, we are conducting this study as part of our Master's in Public Health.* *We want to collect information about SMC campaigns in your village / area.* *With your permission we will ask you a few questions and it will last about twenty minutes.* *Do you agree to participate?* *Yes / - / 2.No / - /*

*Name of Investigator .......................................................................................*

*Date of interview: .......................................................................................*

*Q1.Village / area of ​​...........................................................................*

*Q2.Code / ___ /*

1. Do you know what  SMC is?
2. If so, how did you heard about the intervention ?
3. Did you agree that your child was given the drugs?
4. Have you been shown how you should give your child the medication on the following two days after the first administration by community distributors?
5. Did you administer both doses of medication on the following two days after community health workers gave your child the first dose?
6. What do you think of the SMC drugs?
7. Have you experienced any side effect?
8. If so, what was it and what did you do ?
9. During the campaigns, were you  advise on how to protect your children and yourself against malaria?
10. If so, what have you been said?
11. What is your appreciation of SMC campaigns?
12. What didn’t you like
13. Do you have any recommendations / suggestions to improve the implementation ?

End of interview

Thank you for your participation!

**Information note and Informed Consent Form**

**INFORMATION**

**Introduction**

Good morning, My name is COMPAORE RACHIDATOU and I am a student at the African Institute of Public Health (IASP). We are here to carry out a study as part of our study for obtaining a Master 2 in public health option planning and management of health services. This study is entitled "Evaluation of the of Implementation fidelity of the SMC intervention in the Kaya Health District".

I therefore investigate the implementation of seasonal chemo-prevention in the health district of Kaya. Do you want to learn more about this survey in order to participate? (Interviewer: if 'yes', continue. If 'no', thank and finish the interview.

**Aim of the research**

Malaria remains a real public health problem in Burkina Faso, especially for children under five years of age, and is the leading cause of morbidity and mortality. This study aims to evaluate the implementation of SMC intervention, which has been recognized as an effective strategy to fight malaria in children from 03-59 months in areas with highly seasonal transmission. We hope that the results of this study will provide information to the public, health decision makers and program managers so that they can extend and ensure the sustainability of these appropriate interventions to reduce the burden of morbidity and mortality due to malaria.

**Selection of participants**

You are invited to participate in this research because we believe that the experience of distribution campaigns that you have experienced can go a long way towards improving the process of implementing other nutrition programs.

**Voluntary participation**

Your participation in this study is entirely voluntary. You can stop and withdraw from the interview at any time without any effect on the health services that you or a family member may need in the future. Feel free to let me know if you are no longer willing to continue this interview.

**Proceedings**

We invite you to take part in an interview that will last about 20-30 minutes of your time. This interview will focus on SMC campaign organization, raising awareness about SMC and SMC drug distribution. We are going to ask you questions, and we'll be interested in anything you can say on the subject.

**Confidentiality**

The information you share with us will be kept confidential; It will only be used for the study. The study will use the collective responses and will not reveal the names or any clues that could be linked to the person who gave the information. No person who is not directly involved in this study will be allowed access to the information you give us. If all the results of this study are published, your identity will remain confidential.

There will be no mention of your identity or any statement that may recognize you. The disclosed information will be completely anonymous.

This consent form that contains your name will be kept separate from the questionnaire and will be destroyed within one year.

**Risks and benefits**

Participation in the study does not provide any kind of compensation to participants, but the knowledge you will allow us to acquire may help to take steps to improve the health of children under five years old.

We will maintain the confidentiality of the information, but can not guarantee any violation that may occur. However, questionnaires and files do not contain any personally identifiable information and therefore your participation and information will remain confidential.

**Contact**

This project is led by the African Institute of Public Health (IASP). If you need to contact this research project at a later date, you can contact COMPAORE Rachidatou at 72 37 56 12.

If the information I am giving you is not clear or if you have any questions about this research and this interview, you can ask me now. Do you have any questions?

**CERTIFICATE OF CONSENT**

I have been informed about this research which is about assessing the implementation of SMC in the Kaya Health District. I have read the informed consent form or it has been read to me;I had the opportunity to ask questions about the research and my questions were clearly explained to my satisfaction.

I understand and agree to participate in the study and to permit the use of the transcripts made in this study: Yes / - / No / - /

If yes, my signature or fingerprint below indicates that I understand the purpose, risks and benefits of this study and that I have had answers to my questions. I have been provided with contact information for the one I can contact if I have any questions or complaints. Therefore, I decide to participate voluntarily in the exchanges included in this study.

**Signature or fingerprint** ..................................................................... ..

**Date:** ...... / ...... ../ .......
